# Body composition and aging: cross-sectional results from the INSPIRE study in people 20 to 93 years old

**DOI:** 10.1007/s11357-024-01245-6

**Published:** 2024-07-19

**Authors:** Marguerite Briand, Jeremy Raffin, Emmanuel Gonzalez-Bautista, Patrick Ritz, Gabor Abellan Van Kan, Fabien Pillard, Marie Faruch-Bilfeld, Sophie Guyonnet, Cédric Dray, Bruno Vellas, Philipe de Souto Barreto, Yves Rolland

**Affiliations:** 1 IHU HealthAge, Toulouse, France; 2 Institut du Vieillissement, Centre Hospitalo-Universitaire de Toulouse, Toulouse, France; 3 CERPOP UMR1295, University of Toulouse III, Inserm, UPS, Toulouse, France; 4 Department of Endocrinology, Metabolic Diseases and Nutrition, Toulouse University Hospital, Toulouse, France; 5 Department of Sport Medicine, Hospital and University of Toulouse, Toulouse, France; 6 Institut RESTORE, UMR 1301, University of Toulouse III, Inserm, UPS, CNRS, Toulouse, France; 7 Imaging Department, Hospital and University of Toulouse, Toulouse, France

**Keywords:** Aging, Sarcopenia, Osteoporosis, Body composition, Geroscience

## Abstract

Aging is characterized by several major changes, including altered body composition, which is associated with numerous negative clinical consequences such as sarcopenia, osteoporosis, and frailty. The study is to evaluate body composition parameters depending on age and sex in a population ranging from the young adult to the very old, and to identify break points in the association between body composition and age. In this cross-sectional study, we included the enrolment population of the French INSPIRE-T prospective cohort, accounting for 915 subjects (62% female). Age ranged from 20 to 93 years, median age (years) was 63 (IQR 27). Body composition (lean mass, fat mass, and bone mineral content) was assessed with dual-X-ray absorptiometry (DXA). Different break points in the relationship between age and body composition variables in males and females were identified using a segmented regression analysis adjusted on physical activity, nutritional status, educational level, and comorbidities. Lean mass decreased from the age of 55 years for males (CI 95% 44–66) and 31 years for females (CI 95% 23–39). For fat mass, we observed a trend towards an increase with age for males. For females, we observed an increase with age up to age 75 (CI 95% 62–86), followed by a decreasing trend. In this study, we described the relationship between body composition and age as a function of sex, establishing a foundation for further studies on predictive biomarkers of age-related body composition alteration.

**Supplementary Information:**

The online version contains supplementary material available at 10.1007/s11357-024-01245-6.

## Introduction

Aging is driven by several molecular cellular and physiological changes [1]. This will lead to changes in both body composition (decline lean mass [LM], decline bone mineral content [BMC], and increase fat mass [FM]) and energy metabolism (decline fat-free mass-adjusted daily energy expenditure) [2] which are among the main factors driving the rate of clinical aging [3]. These changes in body composition are associated with multiple negative health outcomes such as sarcopenia or osteoporosis [4, 5], as well as frailty [5] and increased risk of cardiometabolic diseases [6, 7]. For this reason, knowledge of body composition norms as a function of age is a key information as it could enable us to differentiate very early subjects with accelerated or pathological changes in body composition from subjects with changes expected in a context of normal aging. In this respect, body composition parameters could be more accurate indicators of the rate of physiological aging than chronological age. Similarly, knowledge of the normative values of body composition could allow the identification of early biological mechanisms of aging predictive of its alteration, making it possible using biomarkers to initiate early preventive interventions before the appearance of a clinically significant alteration.

The largest studies assessing the association between body composition parameters and age have been conducted in the United States and Australia [8–10] then in Asian countries [11, 12], but the application of the age-specific standards proposed by these studies to other populations such as European populations is questionable, especially considering the differences in lifestyle and dietary patterns [13, 14]. Moreover, few studies include subjects aged 80 years and over [15, 16], especially in the European population where studies ranging from young adults to the very old assessing body composition according to age are scarce with a low proportion of very aged subjects [17, 18]. For example, the largest European study by Ofenheimer et al., with 10,894 patients, does not include patients over 81 years of age [19]. Conversely, other studies that included only elderly patients [20] do not permit the identification of early changes of body composition. Finally, few of these studies have sought to identify break points in the evolution of body composition parameters as a function of age and the findings display substantial variations [15, 21, 22]. There is therefore a need for further studies over a wide age range with standardized measures of body composition.

In this study, we aimed to evaluate three main parameters of body composition: lean mass (LM), fat mass (FM), and bone mineral content (BMC), using dual-energy X-ray absorptiometry (DXA), according to age and sex in a population aged from 20 to 93 years. In addition, we aimed to identify break points associated with body composition changes across the lifespan in males and females.

## Materials and methods

### Study design

We conducted a cross-sectional study using the baseline data of the INSPIRE human translational cohort (INSPIRE-T) [23]. The INSPIRE-T cohort is an observational prospective study (pre-planned 10-year follow-up) that makes part of the broader INSPIRE program, which is an ongoing research initiative dedicated to geroscience that includes both an animal and a human cohort [24]. One of the main objectives of the INSPIRE program is the identification of aging biomarkers using a translational approach from basic biology to the clinical setting [24]. Recruitment for INSPIRE-T took place in the Toulouse area (Southwest, France). Different recruitment strategies were combined, with recruitment via general practitioners, hospital care services, senior residences and retirement homes, and information campaigns in the media [23].

Subjects had to be 20 years or older and affiliated to a health insurance plan. Subjects with a serious condition compromising life expectancy at 5 years were excluded. In the particular case of dependency (defined by the need for assistance with simple acts of daily living), subjects with an estimated life expectancy of less than 1 year were excluded. Individuals under protective measures (curatorship, guardianship, or legal guardianship) were also excluded.

All the patients gave their written informed consent. Full details of the recruitment and sampling strategy are available in the previous publication by Guyonnet et al. [23].

### Measurements

#### Body composition assessment

Body composition was assessed using one single DXA device (GE Healthcare Lunar iDXA) by the same trained examiner. DXA provided values for bone mineral content (BMC), fat mass (FM), and lean mass (LM) [25]. We then calculated different indicators: LM index (LMI = LM/height^2^ in kg/m^2^), FM index (FMI = FM/height^2^ in kg/m^2^)[26]. Appendicular skeletal muscle mass (ASMM in kg) was defined as the sum of the LM of the four limbs and the ASMM index was then calculated (ASMMI = ASMM/height^2^ in kg/m^2^) [27]. The ASMMI was considered low when less than 5.5 kg/m^2^ in females and less than 7 kg/m^2^ in males, following the thresholds for LM recommended by the European Working Group on Sarcopenia in Older People (EWGSOP2) for the diagnosis of sarcopenia [5]; we also calculated the ASMM divided by BMI (ASMM/BMI).

#### Other variables

At the inclusion, we measured height (m) and weight (kg), and BMI was calculated (weight/height^2^ in kg/m^2^). To assess comorbidity burden, a modified version of the Charlson comorbidity index (CCI) was calculated (excluding metastatic solid tumour diagnosis or dementia) [28]. Demographic data were collected as educational level, as well as nutritional status with the mini-nutritional assessment (MNA). The MNA is an 18-item questionnaire, rated from 0 to 30. A nutritional risk was defined by a score less than or equal to 23.5 [29]. Physical performances were assessed using the short physical performance battery (SPPB) ranging from 0 to 12, and SPPB was considered low when below 10 [30]. Physical activity was assessed using the IPAQ long form questionnaire, which provides a quantitative score to estimate the volume of total physical activity in MET-min over 7 days (MET-min/week), and a qualitative score to categorize subjects according to their level of physical activity into three groups: low, moderate, and high [31–33].

### Statistics

Qualitative variables were described in terms of absolute numbers and frequencies and quantitative variables by their median and interquartile range (IQR). Descriptive DXA parameters were presented separately by sex and age group (20–30 years, 30–40 years, 40–50 years, 50–60 years, 60–70 years, 70–80 years, and 80 years and over). The comparison between males and females for quantitative variables were performed using Student’s *t* test for independent samples or the Wilcoxon rank sum test as appropriate. For qualitative variables, we used the Pearson’s chi^2^ test or the Fisher’s exact test as appropriate.

In order to describe the sex-stratified association between the body composition parameters and age, we performed a segmented regression, following the methodology proposed by Pontzer et al. [2, 34]. Briefly, we used the segmented package from R (version 1.6–4). This is an iterative procedure which starts from undertaking a linear regression model that will allow the identification of one or more segmented relationships between two variables and estimating slopes (beta coefficient), as well as breakpoints and their confidence intervals. With this approach, breakpoints are not predefined, but are determined by the model. The complete procedure is described in more detail by Muggeo et al. [34]. We tested different models with 0 to 3 breakpoints. For each body composition variable, we selected the number of breakpoints that would give the best adjusted *R*^2^. When the increase of the number of breakpoints only allowed to identify break points-value that were similar to the previous model, the model with the least number of break points was selected.

Physical activity and nutrition are the two main potential cofounders influencing body composition [35]. Then, to evaluate the intrinsic effect of age, the segmented regression was adjusted on physical activity with the quantitative IPAQ score and nutritional status with the MNA score. The regression was also adjusted on educational level and comorbidities (CCI greater than or equal to 2). Then, the model was adjusted on height for LM and FM. For values already indexed on height such as FMI, LMI, and ASMMI, height was not included in the model. For the BMC, the model was fitted on the IPAQ quantitative score, MNA, BMI, comorbidities, and educational level. Participants with missing data for the covariable included in the model were excluded from the adjusted analysis. Statistical analysis was made using STATA version 18 and R studio version 2022.

## Results

### Description of population

From October 2019 to March 2022, 1015 subjects were included in the INSPIRE T cohort. Among them, the participants of the present study were the 915 subjects who performed a body composition assessment using DXA, being 348 males and 567 females. Their median age was 63 (IQR 48–76) ranging from 20 to 93 years (see supplementary Fig. 1 for flow chart). Males were significantly older. Physical activity estimated in MET-min/week based on the quantitative IPAQ score was higher in men than in women. For the categorical score, the level of physical activity was low for 16.3% of subjects, moderate for 48.4%, and high for 35%. There was no difference between males and females in the proportion of subjects with a low level of physical activity, while the proportion of subjects with a moderate level of physical activity was higher among females, and the proportion of subjects with a high level of physical activity was higher among males.

Males had a significantly higher LM, ASMM, and ASMMI than females (*p* < 0.001). BMC was also higher in males than in females (*p* < 0.001), whereas the FMI was higher in females than in males (*p* < 0.001).

On average in males, between the age groups 20 to 30 and over 80, there was a 11.6% difference in LM (− 6.4 kg), a 35.8% difference in FM (+ 5.8 kg), and a − 3% loss of BMC (− 0.09 kg). In females, there was an 10.8% difference in LM (− 4.2 kg), a 27.2% difference in FM (+ 4.9 kg), and a 22.3% loss of BMC (− 0.52 kg). There was a 5.1% difference in male height (− 9 cm) and 6.1% difference in female height (− 10 cm). For 15 subjects, the IPAQ score was not available (six males, nine females). There was no missing data for the other variables of interest. The full description of the population is available in Table 1, Table 2, and Table 3.

**Table 1:** Characteristic of the population

		*Males*	*Females*	
	*Total*			*p values*
*Population, n (%)*	915	348 (38%)	567 (62%)	
*Age (years)*	63 (28)	67 (27)	61 (27)	< 0.001
*Weight (kg)*	67 (19)	77 (17)	61 (13)	< 0.001
*Height (m)*	1.65 (0.13)	1.74 (0.1)	1.61 (0.1)	< 0.001
*BMI (kg/m*^*2*^*)*	24.5 (2.2)	25.5(4.9)	23.8 (5.2)	< 0.001
* Low BMI (*< *20 if age* < *70 y;* < *22 if age* ≥ *70 y; n (%))*	114 (12.5%)	19 (5.5%)	95 (16.7%)	< 0.001
* Obesity (BMI* > *30, n (%))*	92 (10.0%)	45 (12.9%)	47 (8.3%)	0.023
*LM (kg)*	41.5 (14.4)	53.5 (8.4)	38 (6)	< 0.001
*LMI (LM/height*^*2*^* in kg/m*^*2*^*)*	15.5 (2.9)	17.5 (2)	14.6 (1.7)	< 0.001
*ASMM (kg)*	19.2 (7.5)	25.4 (5.3)	17.2 (3.5)	< 0.001
*ASMMI (kg/m*^*2*^*)*	7.1 (1.7)	8.3 (1.2)	6.6 (1)	< 0.001
*FM (kg)*	21.3 (10.8)	21.1 (10.4)	21.7 (11)	0.194
*FMI (FM/height*^*2*^* in kg/m*^*2*^*)*	7.8 (4.1)	7 (3.5)	8.4 (4.2)	< 0.001
*BMC (kg)*	2.4 (0.9)	3.0 (0.5)	2.1 (0.5)	< 0.001
*CCI* ≥ *2*^*1*^*, n (%)*	72 (7.9%)	39 (11.2%)	33 (5.8%)	0.003
*IPAQ score (MET-min/week)*	2283 (2541)	2580 (2946)	2146 (2247)	< 0.001
*IPAQ categorical score, n (%)*				
* Low physical activity level*	149 (16.3%)	59 (16.9%)	90 (15.9%)	0.667
* Moderate physical activity level*	443 (48.4%)	142 (40.8%)	301 (53.1%)	< 0.001
* High physical activity level*	323 (35%)	147 (42.2%)	176 (31%)	< 0.001
*Low SPPB (SPPB* < *10), n (%)*	50 (5.5%)	16 (4.6%)	34 (6%)	0.366
*MNA (0 to 30)*	28 (2.5)	28 (2)	28 (2.5)	< 0.001
*Nutritional risk (MNA* ≤ *23), n (%)*	41 (4.4%)	11 (3.2%)	30 (5.3%)	0.131

All variables are median (Interquartile range) except specified. *p* values are for comparison between male and female in univariate analysis

*LM* lean mass; *LMI* lean mass index; *ASMM* appendicular skeletal muscle mass; *ASMMI* appendicular skeletal muscle mass index; *BMI* body mass index; *FM* fat mass; *FMI* fat mass index; *BMC* bone mass content; *CCI* Charlson comorbidity index; *IPAQ score* international physical activity quantitative score, with estimation of energy expenditure secondary to physical activity in MET-min/week; *SPPB* short physical performance battery; *MNA* mini-nutritional assessment

**Table 2:** Characteristic of the population in different age groups for males

Age *Years*	Population* n*	Weight *kg*	Height *m*	BMI *kg/m*^*2*^	Nutritional *risk*^*1*^*, n (%)*	Low SPPB^2^ *n (%)*	IPAQ score *MET-min/week*
*20–30*	24	73 (9)	1.78 (0.1)	22.9 (3.1)	2 (8.3)	0 (0)	3481 (3157)
*30–40*	32	80 (16)	1.77 (0.1)	24.7 (5.9)	2 (6.2)	0 (0)	2665 (3650)
*40–50*	27	76 (18)	1.75 (0.1)	25.7 (6.6)	1 (3.7)	0 (0)	3148 (3228)
*50–60*	37	83 (15)	1.80 (0.1)	25.7 (3.8)	2 (5.4)	1 (2.7)	3018 (1896)
*60–70*	68	78 (16)	1.74 (0.1)	25.7 (4.8)	2 (2.9)	2 (2.9)	3341 (3381)
*70–80*	81	79 (19)	1.72 (0.1)	25.9 (4.8)	2 (2.5)	3 (3.7)	2571 (2752)
*80 and over*	79	75 (14)	1.69 (0.1)	25.5 (3.9)	0 (0)	10 (12.7)	1740 (2580)
Age *Years*	Population *n*	LM kg	LMI kg/m^2^	ASMM kg	ASMMI kg/m^2^	FM kg	FMI kg/m^2^
*20–30*	24	55.0 (9)	17.7 (3.1)	26.9 (4.5)	8.87 (1.4)	16.2 (9.6)	4.80 (3.3)
*30–40*	32	56.6 (11.5)	17.6 (3.7)	28.1 (6.4)	8.84 (2.1)	18.6 (13.9)	6.05 (4.7)
*40–50*	27	53.6 (5.9)	17.7 (1.5)	26.1 (3.9)	8.48 (1.0)	21.0 (14)	6.60 (4.6)
*50–60*	37	58.0 (7.3)	18.2 (1.7)	28.4 (3.4)	8.76 (0.9)	21.2 (9)	6.39 (2.6)
*60–70*	68	54.0 (7.1)	17.7 (1.7)	25.7 (5.1)	8.45 (1.3)	20.9 (9.8)	6.85 (3.5)
*70–80*	81	53.2 (7.0)	17.3 (2.3)	24.9 (3.9)	8.11 (1.2)	23.1 (10.7)	7.62 (3.5)
*80 and over*	79	48.6 (7.9)	16.9 (1.8)	22.3 (4.6)	7.79 (0.9)	22.0 (10.3)	7.71 (2.7)

All variables are median (Interquartile range) except specified. *LM* lean mass; *LMI* lean mass index; *ASMM* appendicular skeletal muscle mass; *ASMMI* Appendicular skeletal muscle mass index; *BMI* body mass index; *FM* fat mass; *FMI* fat mass index; *BMC* bone mass content; *IPAQ score* international physical activity quantitative score, with estimation of energy expenditure secondary to physical activity in MET-min/week; *SPPB* short physical performance battery; *MNA* mini-nutritional assessment

^1^MNA ≤ 23.5

^2^SPPB < 10

**Table 3:** Characteristic of the population in different age groups for females

Age *Years*	Population* n*	Weight *kg*	Height *m*	BMI *kg/m*^*2*^	Nutritional *risk*^*1*^*, n (%)*	Low SPPB^2^ *n (%)*	IPAQ score *MET-min/week*
*20–30*	48	60 (11)	1.64 (0.1)	22.3 (3.8)	4 (8.3)	0 (0)	2166 (2349)
*30–40*	52	63 (21)	1.64 (0.1)	23.0 (6.1)	3 (5.8)	0 (0)	2252 (1822)
*40–50*	68	60 (13)	1.63 (0.1)	22.1 (3.7)	3 (4.4)	0 (0)	2067 (2818)
*50–60*	98	63 (12)	1.63 (0.1)	24.0 (4.7)	3 (3.1)	1 (1.02)	2126 (2544)
*60–70*	106	62 (15)	1.60 (0.1)	23.7 (5.5)	5 (4.7)	3 (2.8)	2537 (2143)
*70–80*	107	61 (14)	1.57 (0.1)	24.2 (5.6)	3 (2.8)	9 (8.4)	2189 (2343)
*80 and over*	88	59 (13)	1.54 (0.1)	24.9 (4.5)	9 (10.2)	21 (23.9)	1371 (1562)
Age *Years*	Population *n*	LM kg	LMI kg/m^2^	ASMM kg	ASMMI kg/m^2^	FM kg	FMI kg/m^2^
*20–30*	48	39 (4.5)	14.2 (1.5)	17.9 (2.8)	6.62 (1.01)	18.0 (8.6)	7.0 (3.4)
*30–40*	52	41.2 (5.7)	15.0 (1.4)	19.6 (3.7)	6.98 (1.06)	19.4 (15)	7.0 (5.3)
*40–50*	68	39.4 (5.5)	14.7 (1.7)	18.0 (3.0)	6.69 (0.99)	19.3 (12.1)	7.1 (4.6)
*50–60*	98	39.1 (5.6)	14.8 (1.5)	17.8 (2.5)	6.81 (0.76)	22.8 (10.2)	8.6 (3.8)
*60–70*	106	37.7 (5.4)	14.5 (1.9)	17.2 (3.3)	6.58 (0.97)	21.7 (12.3)	8.3 (4.6)
*70–80*	107	36.6 (6.4)	14.4 (1.7)	16.4 (2.9)	6.52 (0.91)	23.3 (9.6)	9.1 (4.1)
*80 and over*	88	34.8 (6.0)	14.7 (1.7)	14.9 (3.3)	6.27 (1.14)	22.9 (9.4)	9.7 (4.1)

All variables are median (Interquartile range) except specified. *LM* lean mass; *LMI* lean mass index; *ASMM* appendicular skeletal muscle mass; *ASMMI* appendicular skeletal muscle mass index; *BMI* body mass index; *FM* fat mass; *FMI* fat mass index; *BM* bone mass; *IPAQ score* international physical activity quantitative score, with estimation of energy expenditure secondary to physical activity in MET-min/week; *SPPB* short physical performance battery; *MNA* mini-nutritional assessment

^1^MNA ≤ 23.5

^2^ SPPB < 10

### Association between body composition variables and age

**Fig. 1:**
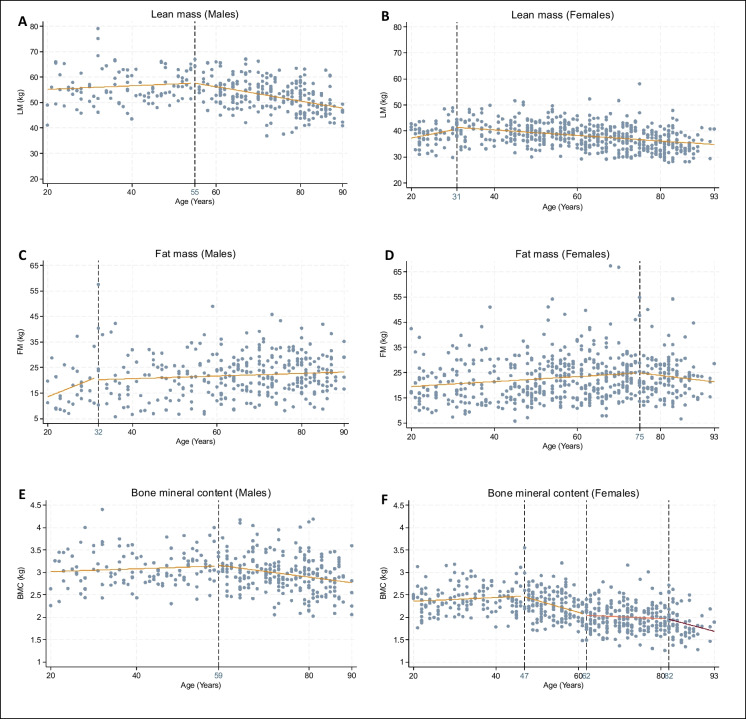
Adjusted segmented regression analysis of LM, FM, and BMC, depending on age, in males and females. LM, lean mass; FM, fat mass; BMC, bone mineral content. Analysis adjusted on: IPAQ quantitative score, MNA, educational level, Charlson comorbidity index (CCI ≥ 2), and height for LM, FM. Analysis adjusted on IPAQ quantitative score, MNA, educational level, Charlson comorbidity index (CCI ≥ 2), and BMI for BM

**Fig. 2:**
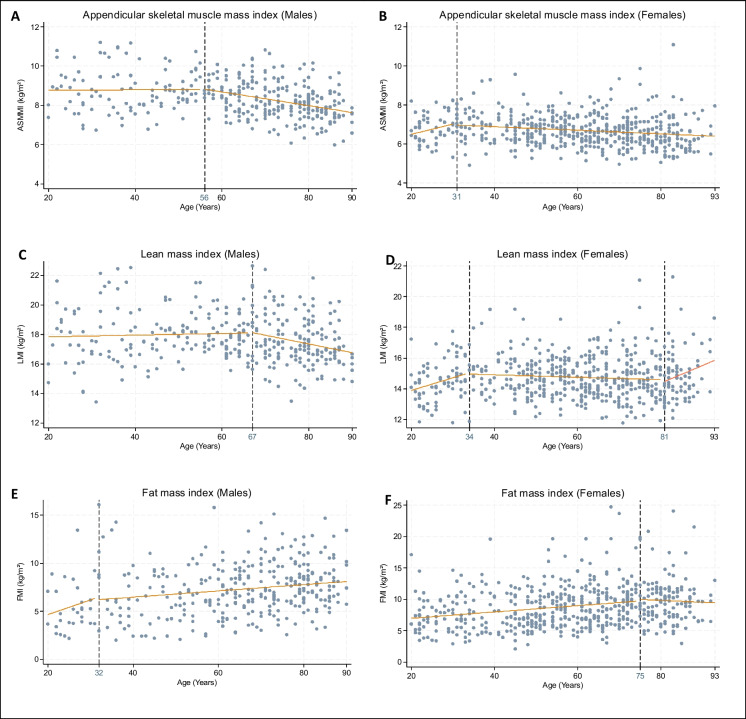
Adjusted segmented regression analysis of ASMMI, LMI, and FMI depending on age, in males and females. ASMMI, appendicular skeletal muscle mass index; LMI, lean mass index; FMI, fat mass index. Analysis adjusted on IPAQ quantitative score, MNA, educational level, Charlson comorbidity index (CCI ≥ 2)

The results of the segmented regression analysis for each body composition parameter are presented in Tables 4 and 5 with their respective graphical representation in Figs. 1 and 2.

**Table 4:** Segmented regression analysis of different body composition variables as a function of age for males

	Unadjusted analysis			Adjusted analysis		
	Break points years (CI 95%)	Coefficient beta (CI 95%)		Break points years (CI 95%)	Coefficient beta (CI 95%)	
*LM* *kg*	55 (47; 63)	20–55 55–93	0.07 (− 0.04; 0.18) − 0.28 (− 0.36; − 0.19)	55 (44; 66)	20–55 55–93	0.04 (− 0.05; 0.14) − 0.16 (− 0.24; − 0.09)
*FM* *kg*	32 (17; 47)	20–32 32–93	0.51 (− 0.41; 1.41) 0.05 (− 0.004; 0.11)	32 (21; 43)	20–32 32–93	0.50 (− 0.22; 1.23) 0.04 (− 0.03; 0.11)
*BMC* *kg*	67 (57; 77)	20–67 67–93	0.001 (− 0.003; 0.01) − 0.01 (− 0.02; − 0.005)	59 (44; 73)	20–59 59–93	0.001 (− 0.003; 0.01) − 0.01 (− 0.02; − 0.005)
*ASMMI kg/m*^*2*^	57 (45; 69)	20–57 57–93	0.0005 (− 0.02; 0.02) − 0.03 (− 0.05; − 0.02)	56 (44; 67)	20–56 56–93	− 0.001 (− 0.02; 0.02) − 0.04 (− 0.05; − 0.02)
*LMI kg/m*^*2*^	67 (56; 78)	20–67 67–93	0.006 (− 0.01; 0.02) − 0.06 (− 0.10; − 0.01)	67 (57; 77)	20–67 67–93	− 0.001 (− 0.02; 0.01) − 0.06 (− 0.11; − 0.02)
*FMI kg/m*^*2*^	32 (5; 59)	20–32 32–93	0.12 (− 0.18; 0.42) 0.03 (0.01; 0.05)	32 (15; 49)	20–32 32–93	0.14 (− 0.15; 0.44) 0.02 (− 0.004; 0.04)

*LM* lean mass, *FM* fat mass, *BMC* bone mineral content, *ASMMI* appendicular skeletal muscle mass index, *LMI* lean mass index, *FMI* fat mass index

Analysis adjusted on: IPAQ quantitative score, MNA, educational level, Charlson comorbidity index (CCI ≥ 2), and height for LM, FM. Analysis adjusted on IPAQ quantitative score MNA, educational level, and Charlson comorbidity index (CCI ≥ 2) for ASMMI, LMI and FMI

Analysis adjusted on IPAQ quantitative score, MNA, educational level, Charlson comorbidity index (CCI ≥ 2), and BMI for BMC

**Table 5:** Segmented regression analysis of different body composition variables as a function of age for females

	Unadjusted analysis			Adjusted analysis		
	Break points years (CI 95%)	Coefficient beta (CI 95%)		Break points years (CI 95%)	Coefficient beta (CI 95%)	
*LM* *kg*	35 (29; 40)	20–35 35–93	0.26 (0.05; 0.47) − 0.12 (− 0.15; − 0.09)	31 (23; 39)	20–31 31–93	0.23 (− 0.05; 0.52) − 0.03 (− 0.05; − 0.01)
*FM* *kg*	74 (62; 86)	20–74 74–93	0.10 (0.05; 0.15) − 0.18 (− 0.51; 0.16)	75 (65; 84)	20–75 75–93	0. 07 (0.02; 0.13) − 0.24 (− 0.59; 0.11)
*BMC* *kg*	47 (42; 51) 63 (58; 68) 78 (70; 85)	20–47 47–63 63–78 78–93	0.004 (− 0.002; 0.01) − 0.03 (− 0.04; − 0.02) − 0.002 (− 0.01;0.01) − 0.02 (− 0.04; − 0.005)	47 (43; 51) 62 (58; 67) 82 (70; 93)	20–47 47–62 62–82 82–93	0.002 (− 0.003; 0.01) − 0.03 (− 0.04; − 0.02) − 0.004 (− 0.01; 0.002) − 0.02 (− 0.05; 0.014)
*ASMMI kg/m*^*2*^	31 (22; 39)	20–31 31–93	0.04 (− 0.02; 0.11) − 0.01 (− 0.01; − 0.005)	31 (23; 39)	20–31 31–93	0.03 (− 0.02; 0.08) − 0.01 (− 0.02; − 0.01)
*LMI kg/m*^*2*^	33 (26; 40) 81 (75;87)	20–33 33–81 81–93	0.08 (0.01; 0.16) − 0.01 (− 0.02; 0.002) 0.09 (− 0.02; 0.20)	34 (26; 42) 81 (77; 85)	20–34 34–81 81–93	0.07 (− 0.002; 0.14) − 0.02 (− 0.03; − 0.005) 0.13 (0.02; 0.23)
*FMI kg/m*^*2*^	75 (53; 97)	20–75 75–93	0.05 (0.03; 0.07) − 0.002 (− 0.13; 0.13)	75 (63; 87)	20–75 75–93	0.03 (0.01; 0.05) − 0.07 (− 0.22; 0.07)

*LM* lean mass, *FM* fat mass, *BMC* bone mineral content, *ASMMI* appendicular skeletal muscle mass index, *LMI* lean mass index, *FMI* fat mass index

Analysis adjusted on: IPAQ quantitative score, MNA, educational level, Charlson comorbidity index (CCI ≥ 2), and height for LM, FM. Analysis adjusted on IPAQ quantitative score, MNA, educational level, Charlson comorbidity index (CCI ≥ 2) for ASMMI, LMI and FMI. Analysis adjusted on IPAQ quantitative score, MNA, educational level, Charlson comorbidity index (CCI ≥ 2), and BMI for BMC

#### Lean mass

After adjustment, lean mass was relatively stable until age 55 in males and age 31 in females. There was then a statistically significant decrease of 0.16 kg/year for males and 0.03 kg/year for females (Fig. 1A–B). For the ASMMI, in males, there was a decreasing trend up to the age of 56 years in males, while it was stable until 31 years of age in females, followed by a significant decrease in both. As for the lean mass, the inflection of the slope seems greater in males than females (− 0.04 kg/years in males versus − 0.01 kg/years in females) (Fig. 2A–B). For LMI, we observed in male a relative stability until the age of 67 years, followed by a significant decrease of 0.06 kg/years. On the contrary, in females we observed a first phase of stability until the age of 34 years, followed by a phase of decrease of 0.02 kg/years, then an increase again from the age of 81 years (Fig. 2C–D). Analyses using ASMM/BMI are shown in **supplementary Materials **Table S1**.**

#### Fat mass

For FM, in males there was tendency to increase with age, initially sharp, then slowing down from age of 32. In females, there was a progressive increase with age, up to 75 years old of 0.07 kg/years, followed by a decreasing trend (Fig. 1C–D). Regarding the FMI, in males there was also a tendency to increase with age, but not statistically significant. In females, there was continuous increase of 0.03 kg/years until 75 years, followed by a downward trend (Fig. 2E–F).

#### Bone mineral content

For males, there was a plateau until the age of 59, followed by a significant decrease in BMC of 0.01 kg/years (Fig. 1E). For females, three break points were identified, with a first stage of stability until the age of 47 years, followed by a rapid decrease of 0.03 kg/years until the age of 62 years. After 62 to 82 years of age, there was a stabilization of the BMC. After 82, there was a downward trend in bone mass (Fig. 1F).

## Discussion

After adjustment on potential cofounders such as physical activity nutritional status comorbidities and educational level, this study identified several break points in the relationship between age and body composition parameters. For LM, we identified a break point at the age of 55 years for males and 31 years for females. For FM, we observed a trend towards an increase with age in males. For females, we observed an increase with age up to 75, followed by a decreasing trend. The identification of break points in our cohort, as reported by others [2], supports the hypothesis that that there are underlying mechanisms at specific periods of life that will play a key role in aging.

For LM, our results were close to the break points found in the Austrian study LEAD, where they identified a decrease of the ASMMI starting from the age group 50–60 years for males, and an increase until the age group 30–40 years then a stabilization for females [19]. In the Geelong osteoporosis study, the inflection of total LM started earlier, between 30 and 39 years in males and 20 and 29 years in females [16], whereas in the Australian body composition (ABC) study, Kirk et al. found a later peak of LM around 50 years [10]. These discrepancies could be explained by the use of a different adjustment method, as well as a different method of recruitment (i.e., in the ABC study, the DXA assessments were performed at the request of the subjects, which could reflect an increased concern for health). Similarly, subjects in our INSPIRE-T cohort may be more likely to monitor their health, which would explain the later decline in LM and ASMMI in males. Although the decline in LM begins around 25 years earlier in females than in males, the slope inflection seems less pronounced than in males, as it has been observed in other studies [19, 22].

Surprisingly, our results for females show an increase in LMI from the age of 81 years, in contrast to our results for ASMMI and LM. This could be explained by the age-related decrease in height, which is more important in females than in males, and can favor an artificial increase in height-indexed variables such as the LMI or the BMI [36]. In our cohort, height was measured with a height gauge the day of the assessment and was not declarative, allowing for an unbiased measure of height. Given a smaller variation of LM with a smaller slope inflection in females than in males, the LMI will be particularly influenced by the decrease in height with age. Indeed, our data showed that women older than 80 years had lower median height than older women at younger ages. In this sense, this index seems to be poorly representative of the modification in muscle mass with age.

For FM, our results are consistent with the literature, with an increase in FM with age in males [9, 10, 22], whereas in females several studies also identified a decrease in FM around the age of 70 to 80 [9, 22].

Our understanding of the mechanisms involved in changes in body composition and of the factors explaining these break points is only partial. The modification of body composition with age can be explained by lifestyle factors (nutrition, physical activity [5]), but also by factors related to the physiological changes conditioning aging [1]. In particular, with aging modifications of energy metabolism, it will be observed as described recently by Pontzer et al. with the identification of inflection ages of energy expenditure adjusted on fat-free mass. Interestingly, in an analysis combining males and females, they observed a decrease in adjusted resting energy expenditure from the age of 46.5 years [2], which is close to the theoretical midpoint of the age of inflection of LM in males and females in our study (respectively 31 and 55 years). Whether the concomitance of break points of energy expenditure and the modification of LM rely on common biological mechanisms remains to be explored.

From a biological point of view, the major changes associated with aging have been determined and updated recently by López-Otín et al. as the hallmarks of aging [1]. In particular, a mechanism of aging associated with energy metabolism that appears to be closely related to alteration of body composition is mitochondrial dysfunction, with decreased efficiency and increased production of reactive oxygen species with aging [1, 37]. Mitochondrial dysfunction is also involved in chronic low-grade inflammation, contributing to changes in body composition with age [38, 39]. In this regard, mitochondrial biomarkers could be predictive of alterations in body composition variables [40, 41]. Similarly, the Apelin, involved in the response to exercise and mitochondriogenesis could be an interesting candidate [42]. Other key mechanisms of aging could also lead to the identification of biomarkers, as actors of nutrient-sensing pathways, epigenetic biomarkers associated with the “epigenetic clock” [1] or biomarkers of the age-related systemic inflammation [43]. Assessment of changes in these biomarkers according to the break points identified by our studies in males and females could lead to a better understanding of the fundamental mechanisms, underlying body composition modifications and the identification of biomarkers predictive of its alterations. The prospective INSPIRE cohort dedicated to geroscience could therefore allow us to monitor these biomarkers, as well as to assess their association with changes in body composition parameters in males and females. Longitudinal studies should investigate the associations of body composition changes with the evolution of functions and the onset of chronic diseases over time.

An advantage of our study is the inclusion of a population ranging from young to very old, with a standardized measurement of body composition by the same operator using the same DXA, allowing assessment at different ages of life. Furthermore, the choice of DXA as a body composition assessment method is a strength of our study as compared to other alternatives, as it allows an accurate, reproducible, and safe assessment of body composition parameters [25, 44]. Another strength of this study is the ability to include elderly and very elderly subjects with different aging trajectories, using a variety of recruitment methods, through the media and primary care practitioners, but also in hospitals, rehabilitation centers, and nursing homes. Finally, we used an innovative statistical analysis in this field, enabling us to identify by an unbiased approach break points for each body composition variable as a function of age.

Nevertheless, our study presents several limitations: Due to its cross-sectional design, we cannot tease out the direction of the association. Secondly, the number of subjects over 90 years of age remains limited which highlights the difficulties of including this population in clinical research. Another limitation is that, in our cohort, a high proportion of subjects displayed a high level of physical activity assessed by the IPAQ score. This could be due to the selection of a more health-conscious population, which suggests that caution should be used in applying our results to a frailer population. High physical activity could also be favored by the subjective nature of the IPAQ score, which can lead to the overestimation of physical activity levels. Also, the use of the MNA to assess nutritional status can be a source of debate, as it is primarily validated in subjects aged 65 and over. However, several studies have also used this score in younger adults [45, 46], and the inclusion in the MNA of items related to dietary patterns, such as protein and dairy product intakes, further increases its relevance to the study of body composition. Finally, as for any observational study, bias related with residual confounding cannot be excluded; for instance, information on sex hormones and menopausal status were not available.

## Conclusion

In this study, by a standardized assessment of body composition, we described the relationship between body composition variables and age and identified different break points for each variable. With this study, we also highlighted the differences related to sex. The INSPIRE cohort gives us the opportunity to continue this work by evaluating the association between changes in biomarkers of aging such as mitochondrial function, epigenetic or inflammatory biomarkers, and the break points of age we identified. Furthermore, studies focusing on the biomarkers associated to body composition changes, as well as mechanistic studies may shed light on the potential mechanisms or targets to avoid major deleterious changes in body composition components.

## Supplementary Information

Below is the link to the electronic supplementary material.

Supplementary file1 (DOCX 26 KB)

## Data Availability

Data described in the manuscript, code book, and analytic code may be made available upon request pending application and approval by the INSPIRE Committee.

## Abbreviations

ASMM: Appendicular skeletal muscle mass
ASMMI: Appendicular skeletal muscle mass index
BMC: Bone mineral content
CCI: Comorbidity Charlson index
DAMPs: Damage-associated molecular pattern
DXA: Dual-X-ray absorptiometry
EWGSOP: European Working Group on Sarcopenia in Older People
IPAQ: International physical activity quantitative score
FM: Fat mass
FMI: Fat mass index
LM: Lean mass
LMI: Lean mass index
MNA: Mini-nutritional assessment
SPPB: Short physical performance battery
