# Supplementary material for: Body composition and aging: cross-sectional results from the INSPIRE study in people 20 to 93 years old
Source: GeroScience. 2024 Jul 19;47(1):863–75. doi: 10.1007/s11357-024-01245-6 (PMC11872965; doi:10.1007/s11357-024-01245-6)
Supplement: Supplementary file 1 — Supplementary file1 (DOCX 26 KB) [file 11357_2024_1245_MOESM1_ESM.docx]

Body composition and aging: cross-sectional results from the INSPIRE study in people 20 to 93 years old.

Marguerite Briand ([briand.m@chu-toulouse.fr](mailto:briand.m@chu-toulouse.fr))

**Supplementary figure 1**. Flow chart

**Excluded**

100 subjects who did not complete a DXA examination.

**Study population**

n = 915

**Inclusion population of the INSPIRE cohort**

Inclusion from 10/2019 to 03/2022

n = 1015

**Supplementary table 1.** Segmented regression analysis of appendicular skeletal muscle mass indexed on BMI as a function of age for males and females.

|  | Unadjusted analysis | | | Adjusted analysis | | |
| --- | --- | --- | --- | --- | --- | --- |
|  | Break points  years (CI95%) | Coefficient beta (CI95%) | | Break points  years (CI95%) | Coefficient Beta (CI 95% ) | |
| Males | 54 (42 ; 66) | 20 – 54  54 – 93 | -0.002 (-0.004; 0.0002)  -0.28 (-0.01 ; -0.005) | 54 (39 ; 69) | 20 – 54  54– 93 | -0.002 (-0.005 ; 0.0004)  -0.16 (-0.008; -0.004) |
| Females | 47 (36; 57) | 20 – 47  47 – 93 | -0.001 (-0.003 ; 0.001)  -0.005 (-0.006 ; -0.004) | 46 (33 ; 59) | 20 – 46  46 – 93 | -0.001 (-0.003 ; 0.001)  -0.004 (-0.005 ; -0.003) |
